# Normal ALT masks occult liver injury in young chronic hepatitis B patients: a call for early treatment intervention

**DOI:** 10.3389/fcimb.2026.1845162

**Published:** 2026-06-02

**Authors:** Pei-pei Wang, Yi Wu, Ting Liu, Ying Zhang, Qing Yang, Xiang-yong Li

**Affiliations:** 1 Department of Infectious Diseases, The Third Affiliated Hospital of Sun Yat-sen University, Guangzhou, Guangdong, China; 2 Guangdong Key Laboratory of Liver Disease Research, The Third Affiliated Hospital of Sun Yat-sen University, Guangzhou, Guangdong, China; 3 Department of Infectious Diseases, The Third Affiliated Hospital of Sun Yat-sen University, Zhaoqing Hospital, Zhaoqing, Guangdong, China; 4 Liver Surgery & Liver Transplantation Center, The Third Affiliated Hospital of Sun Yat-sen University, Guangzhou, Guangdong, China

**Keywords:** antiviral therapy indication, chronic hepatitis B, histological injury, non-invasive model, young patients

## Abstract

**Background:**

Guidelines provide limited guidance on antiviral therapy for CHB patients ≤30 years with normal ALT despite occult injury risks.

**Methods:**

This multicenter cross-sectional study included 423 treatment-naïve CHB patients aged ≤30 years with persistently normal ALT who underwent liver biopsy. Significant injury was defined as METAVIR necroinflammation ≥G2 and/or fibrosis ≥S2. A non-invasive model was developed and internally validated.

**Results:**

Significant necroinflammation and fibrosis were present in 48.0% and 48.5%, respectively. Independent predictors included anti-HBc, LSM, and AST. The Y-HAL model showed excellent discrimination (AUC: 0.918), outperforming 12 existing models. A three-tier pathway stratified patients into low-risk (monitoring), high-risk (antiviral therapy), and intermediate-risk (biopsy) categories.

**Conclusions:**

A substantial proportion of young CHB patients with normal ALT have significant histological injury. The Y-HAL model and three-tier strategy offer an evidence-based framework for early intervention.

## 1 Introduction

Chronic Hepatitis B (CHB) virus infection remains a formidable global health challenge, affecting an estimated 296 million individuals worldwide and causing approximately 820,000 annual deaths, primarily due to cirrhosis and hepatocellular carcinoma (HCC) (Jeng et al., 2023). In highly endemic regions like China (Hou et al., 2020), optimizing the management of the vast existing infected population is paramount (Liu et al., 2019; Yan et al., 2025). The cornerstone of CHB management is the timely initiation of antiviral therapy to suppress viral replication and prevent disease progression (Rajbhandari et al., 2025). Current guidelines from international liver societies (the American Association for the Study of Liver Diseases(AASLD), the European Association for the Study of the Liver (EASL), the Asian Pacific Association for the Study of the Liver (APASL) and Chinese guidelines) provide frameworks for treatment initiation that are largely centered on surrogates of active liver injury, Specifically, elevated alanine aminotransferase (ALT) levels, a high HBV DNA viral load and evidence of advanced fibrosis (Sarin et al., 2016; European Association for the Study of the Liver, 2025; Ghany et al., 2026). Consequently, Hepatitis B e antigen (HBeAg) positive patients under 30 years of age with persistently normal ALT are generally recommended for observation and deferred treatment (You et al., 2023), under the presumption of an immune-tolerant state with minimal liver damage (Sarin et al., 2016; You et al., 2023; Ghany et al., 2026).

However, this reliance on ALT as a gatekeeper for further assessment and therapy is increasingly being questioned (Jiang et al., 2023; Huang et al., 2024). A growing body of evidence from liver biopsy studies consistently demonstrates that a considerable proportion of ALT-normal patients harbor substantial histopathological injury, including moderate-to-severe inflammation and significant fibrosis (Jiang et al., 2023; Li et al., 2023; Gao et al., 2024; Huang et al., 2024). This critical discordance between peripheral biochemical normality and underlying intrahepatic pathology creates a profound clinical dilemma. Adherence to current guidelines risks missing a window for early intervention in young patients who are already experiencing progressive liver injury, potentially allowing fibrosis to advance unchecked over years of supposed “immune tolerance” (Li et al., 2023; Lin et al., 2025).

The central barrier to addressing this issue in routine practice is the invasiveness and limited acceptability of liver biopsy, the gold standard for assessing histology. While non-invasive diagnostic models like FIB-4 and APRI exist, they are primarily derived from and validated in older populations with elevated liver enzymes (Kang et al., 2022). As a result, clinicians are left navigating a significant gray area without robust, evidence-based tools to identify which young, ALT-normal patients require urgent histological evaluation and consideration for antiviral therapy.

To address this gap, we conducted a large-scale, nationwide multicenter study specifically targeting this guideline-ambiguous population. By systematically performing liver biopsies in treatment-naïve CHB patients under 30 with persistently normal ALT, we first sought to definitively quantify the prevalence and severity of this “occult” histological injury. Beyond documenting this clinical reality, our primary aim was to derive and validate a clinically actionable non-invasive model (Y-HAL) that integrates readily available parameters to accurately risk-stratify these patients. Through this work, we advocate for a necessary paradigm shift, moving beyond the sole reliance on ALT, toward a more precise and proactive management strategy for young CHB patients.

## 2 Materials and methods

### 2.1 Patients and study design

This multi-center, cross-sectional human study enrolled treatment-naïve CHB patients with persistently normal ALT levels from nine tertiary hospitals across mainland China between January 2019 and January 2024. Eligible participants met the following inclusion criteria: (1) confirmed hepatitis B surface antigen (HBsAg) positivity for >6 months; (2) age ≤30 years at enrollment; (3) ALT levels persistently within the normal range (≤40 U/L for males, ≤35 U/L for females) on ≥3 consecutive measurements over 6 months period; and (4) willingness to undergo percutaneous liver biopsy without clinical contraindications (e.g., coagulopathy, ascites).

Exclusion criteria comprised: (1) coinfection with hepatitis C virus (HCV), hepatitis D virus (HDV), hepatitis E virus (HEV), or human immunodeficiency virus (HIV); (2) concurrent liver diseases (alcohol-related liver disease, metabolic dysfunction-associated steatotic liver disease (MASLD), autoimmune hepatitis, or inherited metabolic liver disorders); (3) prior antiviral therapy for HBV; (4) pregnancy or lactation; and (5) a family history of HCC or cirrhosis, as these patients already possess distinct, expedited indications for monitoring and intervention outside the clinical ‘gray zone’.

### 2.2 Liver biopsy and histological examination

All enrolled patients underwent ultrasound-guided percutaneous liver biopsy following standardized institutional protocols. Patients were offered the biopsy under an active clinical research protocol designed for proactive risk assessment in patients situated in the clinical ‘gray zone’, given the recognized limitations of ALT as a sole surrogate for occult liver injury.

Liver specimens were obtained using 16-gauge needles. Samples were considered adequate for histopathological evaluation if they met the criteria of ≥2.0 cm in length and contained ≥11 complete portal tracts, ensuring reliability and minimizing sampling error. Specimens were systematically processed with hematoxylin-eosin and Masson’s trichrome staining.

Histological assessments were centrally performed by two blinded expert hepatopathologists from the reference center (Department of Pathology, Sixth Affiliated Hospital of Sun Yat-sen University). To ensure objectivity, pathologists were kept unaware of all clinical and laboratory data. Liver inflammation and fibrosis were graded according to the METAVIR scoring system. Clinically significant necroinflammation was defined as grade≥G2, and significant fibrosis was defined as stage≥S2. These METAVIR thresholds were selected as they represent widely accepted, clinically critical inflection points for disease progression and are established by international guidelines as primary indications for initiating antiviral therapy. Interobserver agreement was evaluated using Cohen’s kappa statistic. Discrepancies (kappa <0.9) were resolved through consensus review by a third senior hepatopathologist. Interobserver agreement was evaluated using Cohen’s kappa statistic. Discrepancies (kappa <0.9) were resolved through consensus review by a third senior hepatopathologist.

### 2.3 Data collection and laboratory examination

Demographic, clinical, and laboratory parameters were systematically collected within two weeks prior to liver biopsy. Clinical parameters included body mass index (BMI) and liver stiffness measurement (LSM) assessed via Fibroscan (Echosens, France).

Laboratory assessments encompassed Haematology (White blood cell count (WBC), platelet count (PLT), hemoglobin (HB), Neutrophils (Neu)) and Liver biochemistry (ALT, aspartate aminotransferase (AST), alkaline phosphatase (ALP), γ-glutamyl transpeptidase (GGT), albumin (ALB), globulin (GLB), total bilirubin (TBIL), and prothrombin time activity (PTA), international normalized ratio (INR)). Virologic profiling included quantitative hepatitis B surface antigen (qHBsAg) and anti-hepatitis B core antibody (anti-HBc) levels were measured using chemiluminescent microparticle immunoassays (ARCHITECT i2000SR, Abbott Laboratories) and sandwich ELISA (Wantai Biological Pharmacy, China), respectively. Serum HBV DNA quantification was performed via COBAS AmpliPrep/COBAS TaqMan v2.0 (Roche Diagnostics), with a lower detection limit of 10 IU/mL. Serum α-fetoprotein (AFP) was quantified via electrochemiluminescence immunoassay. Virologic markers (qHBsAg, HBV DNA) were log10-transformed for analysis. Data collection adhered to standardized protocols across participating centers to minimize inter-institutional variability.

### 2.4 Ethics statement

This human study, which involved invasive percutaneous liver biopsies, was conducted in strict accordance with the principles of the Declaration of Helsinki. The study protocol was approved by the Research Ethics Committee of the Third Affiliated Hospital of Sun Yat-sen University (Approval Number: II2023-150-01). Written informed consent was obtained from all individual participants prior to enrollment and biopsy procedures.

### 2.5 Statistical analysis

All statistical analyses were performed using R software (version 4.2.1). Continuous variables were expressed as medians with interquartile ranges (IQR) and compared using the Mann-Whitney U test or Kruskal-Wallis test, as appropriate. Categorical variables were presented as numbers (percentages) and compared using the Chi-square test or Fisher’s exact test.

For model development and internal validation, the study cohort was randomly split into a training set and an internal validation set at a 7:3 ratio. Univariate logistic regression analysis was first performed to identify variables associated with significant histological injury (inflammation grade ≥G2 and/or fibrosis stage ≥S2). Variables with a p-value < 0.05 first underwent collinearity diagnosis. Those with a variance inflation factor (VIF) of < 5 were entered into a multivariate logistic regression model using a stepwise backward selection procedure to identify independent predictors and construct the final Y-HAL model.

The model’s discriminative ability was evaluated using receiver operating characteristic (ROC) curves and the area under the curve (AUC). To comprehensively report model performance, sensitivity, specificity, positive predictive value (PPV), negative predictive value (NPV), and overall accuracy were calculated at optimal cut-offs. Model calibration, assessing the agreement between predicted probabilities and observed outcomes, was evaluated using calibration curves and Spiegelhalter’s Z-test.

The discriminative ability of the Y-HAL model was compared with 12 existing non-invasive models using DeLong’s test. A two-tailed *P*-value < 0.05 was considered statistically significant.

## 3 Results

### 3.1 Study population and baseline characteristics

Between January 2019 and January 2024, a total of 471 treatment-naïve CHB patients aged ≤30 years with persistently normal alanine aminotransferase (ALT ≤40 U/L) were prospectively enrolled across nine tertiary centers in China. After excluding 48 individuals (14 for alcohol use, 16 who declined liver biopsy, and 18 with a family history of hepatocellular carcinoma or cirrhosis), 423 patients were included in the final analysis. The patient selection process is detailed in **Supplementary Figure 1**.

Baseline demographic, clinical, and laboratory characteristics of the total cohort are summarized in **Table 1**. The study population had a median age of 29.0 years (IQR: 27.0–30.0) and was predominantly male (61.2%, 259/423). As per the inclusion criteria, liver biochemistry parameters were within the normal range, with a median ALT of 27.0 U/L (IQR: 19.0–36.6). The median platelet count was 204 ×10^9^/L (IQR: 173–238). Virologically, the majority of patients were hepatitis B e antigen (HBeAg)-positive (82.0%, 347/423), with a median HBV DNA load of 4.32 log^10^ IU/mL (IQR: 2.89–7.46). LSM indicated a median value of 6.40 kPa (IQR: 5.10–8.70), and AFP levels were within normal limits (median: 2.58 ng/mL, IQR: 1.87–4.04).

**Table 1:** Baseline characteristics of the total study cohort.

Variables	Total (N = 423)	Center 1 (N = 54)	Center 2 (N = 35)	Center 3 (N = 65)	Center 4 (N = 29)	Center 5 (N = 56)	Center 6 (N = 43)	Center 7 (N = 11)	Center 8 (N = 29)	Center 9 (N = 101)
Age, y	29.0 [27.0-30.0]	29.0 [27.0-30.0]	29.0 [26.0-30.0]	29.0 [28.0-29.0]	28.0 [28.0-29.0]	29.0 [28.0-29.0]	28.0 [25.0-29.5]	28.0 [28.0-30.0]	27.0 [18.0-29.0]	30.0 [28.0-30.0]
Male, n (%)	259 (61.2%)	31 (57.4%)	23 (65.7%)	48 (73.8%)	13 (44.8%)	33 (58.9%)	18 (41.9%)	9 (81.8%)	16 (55.2%)	68 (67.3%)
WBC (10^9^/L)	5.91 [4.88-6.94]	5.76 [4.72-7.23]	5.92 [4.84-7.14]	6.36 [5.36-7.49]	5.99 [5.14-6.67]	5.94 [5.15-6.91]	5.63 [4.36-6.38]	6.09 [5.76-6.69]	6.57 [5.84-7.34]	5.59 [4.80-6.63]
RBC (10^9^/L)	4.82 [4.44-5.20]	4.76 [4.31-5.10]	4.94 [4.72-5.33]	4.80 [4.39-5.23]	4.69 [4.41-5.18]	4.83 [4.39-5.06]	4.63 [4.19-4.86]	5.22 [4.94-5.54]	4.90 [4.48-5.23]	4.88 [4.61-5.28]
HGB (g/dL)	14.4 [13.2-15.5]	14.3 [13.0-15.0]	15.0 [14.0-15.9]	14.7 [13.6-15.3]	13.7 [12.9-15.0]	14.6 [13.5-15.8]	13.6 [12.6-15.4]	14.5 [13.6-15.5]	13.7 [12.9-15.2]	14.6 [13.5-15.5]
PLT (10^9^/L)	204 [173-238]	192 [154-229]	192 [171-225]	197 [163-223]	231 [204-268]	202 [168-245]	186 [166-225]	202 [172-256]	206 [196-218]	219 [179-248]
AST (U/L)	24.0 [20.0-30.0]	22.0 [19.0-28.0]	23.0 [19.0-27.0]	25.0 [21.0-33.0]	31.0 [20.0-35.0]	21.8 [18.0-27.0]	20.0 [17.0-22.5]	23.0 [19.0-27.5]	21.5 [18.1-25.4]	27.0 [23.0-33.0]
ALT (U/L)	27.0 [19.0-36.6]	23.0 [16.0-35.0]	27.0 [22.0-34.0]	33.0 [23.0-42.0]	26.0 [23.0-30.0]	21.7 [15.3-31.1]	20.0 [15.4-25.6]	24.0 [21.5-27.0]	25.1 [17.9-35.9]	32.0 [23.0-44.0]
ALB (g/L)	45.0 [42.2-47.4]	44.2 [42.0-46.6]	42.0 [41.0-45.0]	46.2 [42.8-48.2]	22.0 [18.0-28.0]	44.8 [43.2-46.4]	45.2 [43.1-46.7]	45.0 [44.1-47.8]	43.0 [40.2-45.0]	47.3 [45.0-49.3]
GLB (g/L)	27.6 [25.0-30.4]	25.8 [23.0-27.5]	28.8 [26.0-30.1]	27.9 [25.2-30.1]	75.1 [69.4-78.5]	27.4 [24.9-30.4]	28.9 [26.9-31.1]	29.2 [26.9-32.0]	26.5 [24.0-29.0]	26.3 [24.4-29.4]
TB (μmol/L)	12.4 [9.45-17.4]	12.3 [8.68-16.7]	11.8 [9.55-14.9]	11.8 [9.90-15.9]	45.7 [43.5-46.9]	11.6 [9.07-16.1]	15.0 [9.95-20.6]	14.9 [8.65-17.9]	10.1 [8.62-13.4]	11.7 [9.30-15.7]
DB (μmol/L)	4.40 [3.34-6.30]	3.70 [2.65-5.42]	4.90 [4.20-6.15]	4.90 [3.80-6.20]	28.0 [25.0-31.1]	3.70 [3.08-5.12]	5.90 [4.50-7.85]	3.90 [3.25-4.75]	3.89 [3.37-4.85]	4.10 [3.10-5.70]
ALP (U/L)	66.0 [55.0-82.5]	67.5 [57.5-83.8]	67.0 [58.5-75.0]	70.0 [59.0-84.0]	13.8 [9.90-18.0]	71.3 [59.2-87.8]	63.0 [56.5-75.0]	62.0 [54.5-75.5]	64.0 [56.0-78.0]	69.0 [57.0-83.0]
GGT (U/L)	18.0 [13.0-26.5]	16.5 [11.2-28.2]	15.0 [13.5-18.5]	25.0 [17.0-37.0]	3.90 [3.00-6.10]	17.6 [12.7-25.0]	14.0 [12.0-19.5]	18.0 [16.5-21.5]	15.0 [12.0-18.0]	22.0 [17.0-32.0]
PT (s)	13.1 [11.8-13.8]	11.2 [10.7-11.9]	13.6 [13.2-14.1]	11.6 [11.0-12.2]	11.7 [11.2-12.2]	13.2 [12.6-13.6]	13.4 [13.0-13.9]	12.6 [12.4-13.0]	13.4 [13.2-13.8]	13.8 [13.4-14.2]
INR	1.02 [0.97-1.07]	1.02 [0.97-1.08]	1.04 [1.00-1.08]	0.98 [0.93-1.03]	1.00 [0.95-1.06]	1.00 [0.96-1.04]	1.01 [0.98-1.06]	0.95 [0.91-1.00]	1.02 [1.00-1.07]	1.06 [1.02-1.10]
HBsAg, log_10_(IU/ml)	3.40 [3.02-3.80]	3.20 [3.01-3.37]	3.76 [3.21-4.64]	3.76 [3.45-3.82]	3.67 [2.40-4.44]	2.93 [1.76-3.40]	3.56 [3.35-3.80]	2.35 [2.10-3.41]	3.37 [2.40-3.40]	3.49 [3.20-3.83]
HBsAb Positive, n(%)	28 (6.62%)	3 (5.56%)	0 (0.00%)	11 (16.9%)	2 (6.90%)	1 (1.79%)	0 (0.00%)	0 (0.00%)	1 (3.45%)	10 (9.90%)
HBeAg Positive, n(%)	347 (82.0%)	37 (68.5%)	35 (100%)	48 (73.8%)	27 (93.1%)	38 (67.9%)	43 (100%)	1 (9.09%)	23 (79.3%)	95 (94.1%)
HBeAb Positive, n(%)	237 (56.0%)	16 (29.6%)	21 (60.0%)	41 (63.1%)	22 (75.9%)	38 (67.9%)	14 (32.6%)	10 (90.9%)	16 (55.2%)	59 (58.4%)
HBcAb,(IU/ml)	3.72 [0.96-7.00]	1.01 [0.70-7.00]	3.55 [1.06-9.68]	5.00 [1.50-7.07]	7.40 [3.45-8.60]	5.74 [1.55-7.19]	1.05 [0.88-7.23]	0.63 [0.61-3.76]	7.00 [5.00-7.00]	3.45 [0.93-6.00]
Inflammation grade(G0/G1/G2/G3/G4)	1/219/133/59/11	0/38/12/4/0	0/24/10/1/0	0/23/12/27/3	0/9/20/0/0	0/13/27/10/6	0/39/3/1/0	0/8/1/2/0	0/5/20/4/0	1/60/28/10/0
Fibrosis stage (S0/S1/S2/S3)	24/194/125/51/29	0/42/5/3/4	0/19/8/6/2	0/29/22/11/3	0/15/13/1/0	1/12/24/7/12	0/29/5/5/4	1/7/2/0/1	0/7/18/4/0	22/34/28/14/3
AFP (ng/mL)	2.58 [1.87-4.04]	2.83 [2.00-4.16]	2.80 [2.08-4.38]	3.22 [1.97-4.84]	2.82 [2.00-3.55]	2.63 [1.75-5.33]	2.06 [1.62-2.84]	2.30 [1.89-3.18]	1.93 [1.66-3.42]	2.55 [1.85-3.96]
LSM(KPa)	6.40 [5.10-8.70]	5.60 [4.60-6.97]	6.40 [5.35-7.75]	6.60 [5.10-8.10]	5.50 [4.40-6.60]	9.25 [7.62-12.6]	5.90 [4.45-7.30]	7.20 [5.55-9.07]	5.80 [4.60-8.00]	6.60 [5.80-8.50]
HBV DNA, log10(IU/ml)	4.32 [2.89-7.46]	3.64 [2.23-7.35]	6.94 [3.27-8.00]	6.00 [4.15-7.32]	5.91 [3.51-7.68]	3.05 [1.96-5.24]	3.29 [2.26-4.01]	3.00 [3.00-3.27]	3.85 [3.23-6.36]	5.59 [3.45-7.96]

Data are presented as median [interquartile range] for continuous variables and n (%) for categorical variables. Hemoglobin (HGB) values are expressed in g/dL. WBC, white blood cell count; RBC, red blood cell count; HGB, hemoglobin; PLT, platelet count; AST, aspartate aminotransferase; ALT, alanine aminotransferase; ALB, albumin; GLB, globulin; TB, total bilirubin; DB, direct bilirubin; ALP, alkaline phosphatase; GGT, gamma-glutamyl transferase; PT, prothrombin time; INR, international normalized ratio; HBsAg, hepatitis B surface antigen; HBsAb, hepatitis B surface antibody; HBeAg, hepatitis B e antigen; HBeAb, hepatitis B e antibody; HBcAb, hepatitis B core antibody; AFP, alpha-fetoprotein; LSM, liver stiffness measurement; HBV, hepatitis B virus.

### 3.2 Normal ALT masks prevalent and clinically significant histological injury

Histopathological evaluation uncovered a critical discordance between biochemical normality and underlying liver injury in this young, ALT-normal CHB cohort. To ensure robustness, significant necroinflammation and significant fibrosis were analyzed both collectively and separately. Significant necroinflammatory activity (≥G2) was identified in 48.0% (203/423) of patients, comprising G2 in 31.4% (133/423) and G3-G4 in 16.5% (70/423). Regarding fibrosis, 48.5% (205/423) of patients exhibited significant fibrosis (≥S2), comprising S2 in 29.6% (125/423), S3 in 12.1% (51/423), and cirrhosis (S4) in 6.9% (29/423). The detailed distribution of inflammation grades and fibrosis stages, visually presented in **Supplementary Figure 2**, confirms the heterogeneity and severity of liver damage within this population.

Comparative analysis between patients with (N = 242) and without (N = 181) significant injury identified key parameters associated with this occult disease (**Table 2**). Patients with significant injury had markedly higher liver stiffness (LSM: 7.35 vs. 5.80 kPa, *P* < 0.001) and anti-HBc levels (7.00 vs. 0.90 IU/mL, *P* < 0.001). They also exhibited higher levels of AST (25.1 vs. 22.0 U/L, *P* < 0.001) and GGT (18.5 vs. 17.0 U/L, *P* = 0.005), alongside lower platelet counts (201 vs. 210 ×10^9^/L, *P* = 0.040) and albumin levels (44.5 vs. 46.0 g/L, *P* < 0.001). Crucially, ALT levels were comparable between the groups (28.0 vs. 25.0 U/L, *P* = 0.088), and no significant differences were observed in virological markers such as HBsAg, HBeAg status, or HBV DNA level, underscoring the limitation of relying on these conventional parameters for treatment decisions.

**Table 2:** Comparative analysis of patients with and without significant histological injury.

Variables	Total N=423	G0/G1 and/or S0/S1 N=181	≥G2 and/or ≥S2 N=242	*P* value
Age, y	29.0 [27.0-30.0]	29.0 [27.0-30.0]	29.0[27.0-29.0]	0.059
Male gender, n(%)	259 (61.2%)	104 (57.5%)	155 (64.0%)	0.202
WBC(10^9^/L)	5.91 [4.88-6.94]	5.89 [4.78-6.84]	5.94 [5.04-7.01]	0.359
RBC(10^9^/L)	4.82 [4.44-5.20]	4.79 [4.41-5.22]	4.84 [4.46-5.19]	0.659
HGB(g/dL)	14.4 [13.2-15.5]	14.4 [13.0-15.3]	14.5 [13.4-15.6]	0.152
PLT(10^9^/L)	204 [173-238]	210 [177-246]	201 [170-230]	0.040
AST(U/L)	24.0 [20.0-30.0]	22.0 [19.0-27.0]	25.1 [20.0-32.0]	<0.001
ALT(U/L)	27.0 [19.0-36.6]	25.0 [18.0-34.0]	28.0 [19.0-37.9]	0.088
ALB(g/L)	45.0 [42.2-47.4]	46.0 [43.0-48.3]	44.5 [41.8-46.8]	<0.001
GLB(g/L)	27.6 [25.0-30.4]	27.4 [24.8-29.9]	28.0 [25.3-30.9]	0.079
TB(μmol/L)	12.4 [9.45-17.4]	12.4 [9.80-17.4]	12.4 [9.17-17.2]	0.602
DB(μmol/L)	4.40 [3.34-6.30]	4.60 [3.30-6.00]	4.30 [3.38-6.35]	0.971
ALP(U/L)	66.0 [55.0-82.5]	68.0 [55.0-80.0]	65.0 [56.0-84.0]	0.853
GGT(U/L)	18.0 [13.0-26.5]	17.0 [12.0-22.0]	18.5 [13.0-32.0]	0.005
PT(s)	13.1 [11.8-13.8]	13.0 [11.4-13.6]	13.2 [12.1-13.8]	0.074
INR	1.02 [0.97-1.07]	1.01 [0.96-1.06]	1.03 [0.98-1.08]	0.032
HBsAg log_10_(IU/ml)	3.40 [3.02-3.80]	3.40 [3.06-3.85]	3.40 [3.01-3.78]	0.439
HBsAb Positive, n(%)	28 (6.62%)	7 (3.87%)	21 (8.68%)	0.077
HBeAg Positive, n(%)	347 (82.0%)	145 (80.1%)	202 (83.5%)	0.446
HBeAb Positive, n(%)	237 (56.0%)	102 (56.4%)	135 (55.8%)	0.986
HBcAb (IU/ml)	3.72 [0.96-7.00]	0.90 [0.76-1.50]	7.00 [3.96-8.27]	<0.001
HBV DNA log10 (IU/ml)	4.32 [2.89-7.46]	4.36 [2.79-7.89]	4.30 [2.93-6.89]	0.106
AFP (ng/mL)	2.58 [1.87-4.04]	2.59 [1.91-3.86]	2.56 [1.80-4.22]	0.771
LSM(KPa)	6.40 [5.10-8.70]	5.80 [4.60-7.10]	7.35 [5.60-10.3]	<0.001

Data are presented as median [interquartile range] for continuous variables and n (%) for categorical variables. Significant histological injury was defined as necroinflammatory activity grade ≥G2 and/or fibrosis stage ≥S2. Hemoglobin (HGB) values are expressed in g/dL. *P*-values were calculated using the Mann-Whitney U test for continuous variables and the Chi-square test for categorical variables. A two-tailed *P*-value <0.05 was considered statistically significant. WBC, white blood cell count; RBC, red blood cell count; HGB, hemoglobin; PLT, platelet count; AST, aspartate aminotransferase; ALT, alanine aminotransferase; ALB, albumin; GLB, globulin; TB, total bilirubin; DB, direct bilirubin; ALP, alkaline phosphatase; GGT, gamma-glutamyl transferase; PT, prothrombin time; INR, international normalized ratio; HBsAg, hepatitis B surface antigen; HBsAb, hepatitis B surface antibody; HBeAg, hepatitis B e antigen; HBeAb, hepatitis B e antibody; HBcAb, hepatitis B core antibody; AFP, alpha-fetoprotein; LSM, liver stiffness measurement; HBV, hepatitis B virus.

### 3.3 Liver injury severity appears independent of HBeAg status in the current cohort

We further investigated whether the drivers of significant injury differed by viral activity status by stratifying the 242 patients with significant histological injury (≥G2 and/or ≥S2) based on their HBeAg status. As shown in **Supplementary Table 1**, the vast majority of these patients were HBeAg-positive (83.5%, 202/242), while a smaller yet substantial subgroup was HBeAg-negative (16.5%, 40/242).

While virological profiles of both HBV DNA (median: 3.00 log^10^ IU/mL vs. 4.60 log^10^ IU/mL in HBeAg-positive patients, *P* < 0.001) and quantitative HBsAg (median: 3.24 log^10^ IU/mL vs. 3.40 log^10^ IU/mL, *P* = 0.009) differed significantly between the two subgroups as expected, key markers directly associated with the severity of liver injury demonstrated no statistically significant difference. Most notably, liver stiffness measurement (LSM: 7.70 kPa vs. 7.30 kPa, *P* = 0.680) and anti-HBc levels (7.00 IU/mL vs. 6.99 IU/mL, *P* = 0.426) were comparable. Additionally, AST, ALT, platelet count, and albumin levels all showed no statistically significant differences (all *P >*0.05) (**Supplementary Figure 3**).

However, given the relatively small sample size of the HBeAg-negative subgroup (N = 40), these findings should be interpreted with caution. The lack of statistical significance may be susceptible to Type II error, and further large-scale prospective studies are necessary to definitively ascertain whether occult injury patterns are truly independent of HBeAg status in young, ALT-normal patients.

### 3.4 A novel model integrating HBcAb, LSM and AST accurately predicts histological injury

Given the high prevalence of occult liver injury undetectable by routine tests, we developed a practical tool to identify at-risk patients. The original cohorts were randomized into a training set (N = 297) for model development and a validation set (N = 126) for internal validation (**Supplementary Figure 1**).

Univariate analysis identified several variables associated with significant histological injury. Among these, HBcAb (OR = 1.78, 95% CI: 1.58-2.01, *P* < 0.001), LSM (OR = 1.30, 95% CI: 1.17-1.44, *P* < 0.001), and AST (OR = 1.06, 95% CI: 1.03-1.09, *P* < 0.001) demonstrated strong associations. The final parsimonious multivariate model confirmed all three as robust, independent predictors: HBcAb level (adjusted Odds Ratio [aOR] = 1.84, 95% CI: 1.62–2.14, *P* < 0.001), liver stiffness measurement (LSM) (aOR = 1.38, 95% CI: 1.20–1.60, *P* < 0.001), and AST level (aOR = 1.06, 95% CI: 1.02–1.10, *P* = 0.009) (**Supplementary Table 2**).

**Figure 1:**
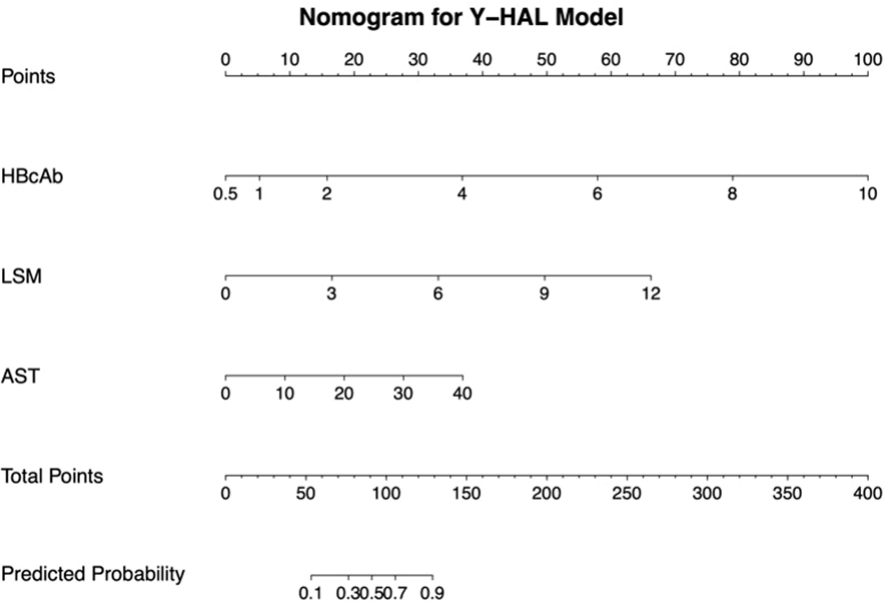
Nomogram of the Y-HAL model for predicting the probability of significant histological injury. The nomogram was developed based on the training cohort (N = 297). It integrates three routinely available parameters: anti-hepatitis B core antibody (anti-HBc, IU/mL), liver stiffness measurement (LSM, kPa), and aspartate aminotransferase (AST, U/L). Points assigned for each variable are summed to obtain a total points score, which corresponds to a predicted probability of significant injury. Significant injury is defined as METAVIR necroinflammation grade ≥G2 and/or fibrosis stage ≥S2.

The model demonstrated excellent and stable discriminative ability, yielded an area under the receiver operating characteristic curve (AUC) of 0.912 (95% CI: 0.897-0.945). in the training set and maintaining a high AUC of 0.929 (95% CI: 0.885-0.974) in the validation set (**Supplementary Figure 4**). Calibration curves demonstrated excellent agreement between predicted probabilities and actual observed frequencies (**Supplementary Figure 5**). As for diagnostic performance metrics, at the optimal cut-off values is detailed in **Supplementary Table 3**. In the validation set, at a cut-off of 0.464, the model achieved a sensitivity of 93.2%, a specificity of 83.0%, a positive predictive value (PPV) of 88.3%, and a negative predictive value (NPV) of 89.8%, with an overall accuracy of 88.9%. For straightforward clinical application, a nomogram was created to visualize the model (**Figure 1**).

### 3.5 The Y-HAL model outperforms existing tools and enables straightforward clinical decision-making

**Figure 2:**
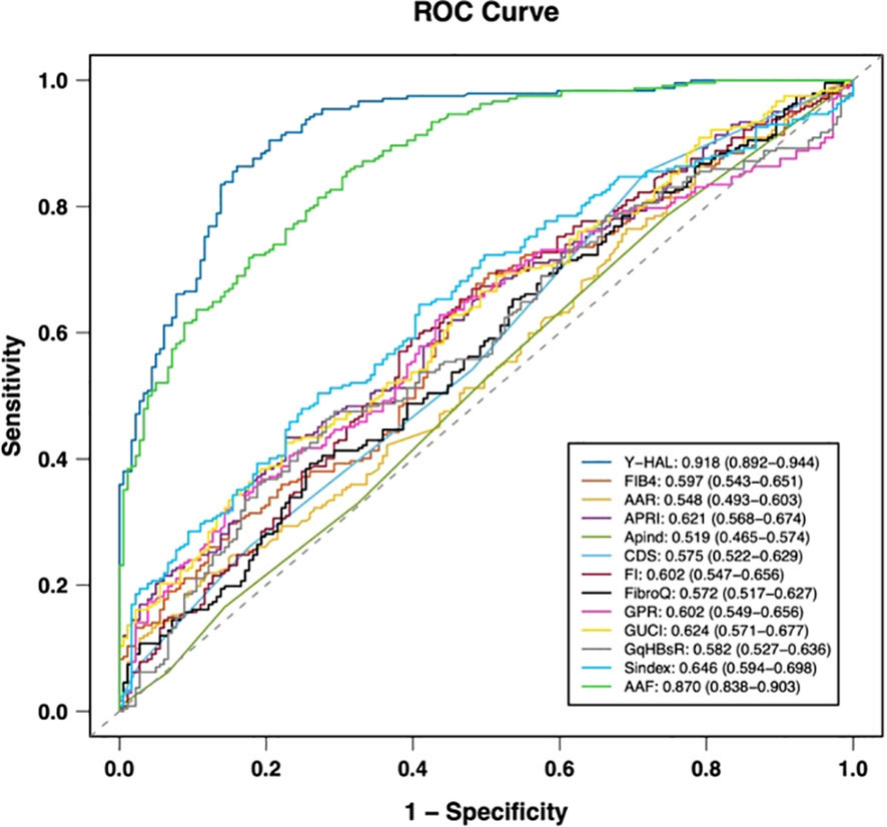
Comparison of the Y-HAL model with established non-invasive models for identifying significant histological injury. Receiver operating characteristic (ROC) curves of the Y-HAL model and 12 previously published non-invasive models in the total cohort (N = 423). The Y-HAL model achieved a superior area under the curve (AUC) of 0.918 (95% CI: 0.892–0.944). ROC, receiver operating characteristic; AUC, area under the curve; CI, confidence interval; FIB-4, Fibrosis-4 index; AAR, AST/ALT ratio; APRI, AST to Platelet Ratio Index; Apind, Age-platelet index; CDS, cirrhosis discriminant score; FI, Fibrosis Index; GPR, gamma-glutamyl transpeptidase to platelet ratio; GUCI, Göteborg University Cirrhosis Index; GqHBsR, Hepatitis B s-antigen quantitative relation; FibroQ, Fibro-Q score.

To contextualize its performance, we conducted a head-to-head comparison of our model against 12 established non-invasive models (e.g., FIB-4, APRI, AAR) within the total cohort. As summarized in **Supplementary Table 4** and visually presented in **Figure 2**, our model (termed Y-HAL) significantly outperformed all existing alternatives. It achieved a superior AUC of 0.918 (95% CI: 0.892-0.944) in the total set, substantially higher than the next best performer (AAF, AUC = 0.870) and far exceeded the commonly used tests like FIB-4 (AUC = 0.597) and APRI (AUC = 0.621).

The final Y-HAL model is defined by the following equation: M = 0.612 × HBcAb (IU/ml) + 0.321 × LSM (kPa) + 0.054 × AST (U/L) - 5.6. The resultant probability is calculated as: Y-HAL Index = exp(M)/[1 + exp(M)].

To translate this superior performance into a practical clinical framework, we established two critical cut-off values (0.21 and 0.87) to prioritize clinical safety and precision. The lower cut-off of 0.21 was selected to prioritize high sensitivity and maximize the negative predictive value (NPV). At this threshold, the model achieved a sensitivity of 97.10% and an NPV of 94.10%. This reliably identifies a low-risk population (27.9% of the cohort) in which the proportion of missed significant injury is minimal (5.9%, 7/118). Conversely, the upper cut-off of 0.87 was chosen to guarantee high specificity and positive predictive value (PPV). At this threshold, the model achieved a specificity of 95.00% and a PPV of 93.70%. This securely defines a high-risk group (33.6% of the cohort) where over 93% of patients are confirmed to have significant injury, firmly justifying treatment initiation.

**Figure 3:**
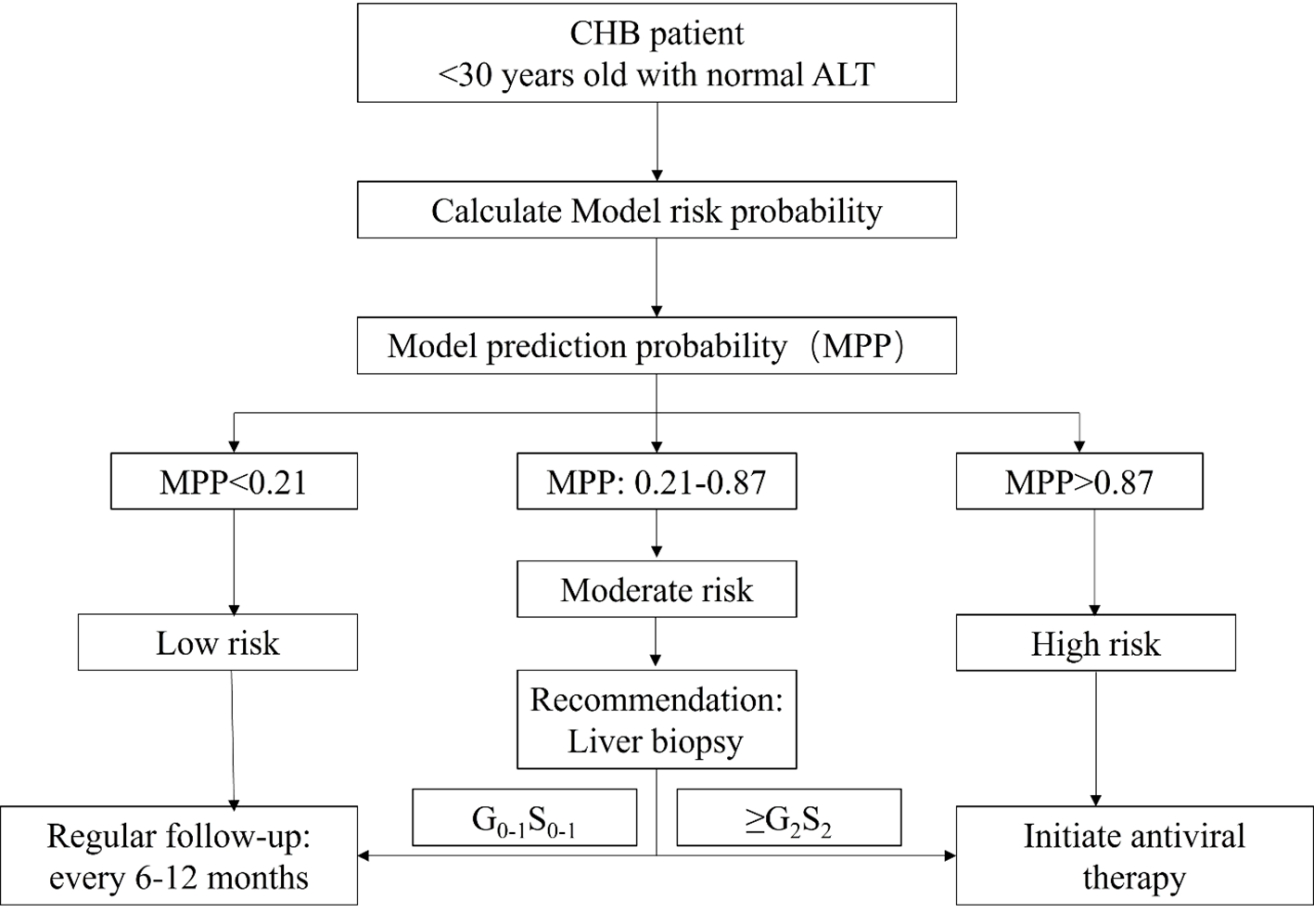
Proposed clinical decision pathway for antiviral therapy initiation in CHB patients aged <30 years with persistently normal ALT. After calculating the Y-HAL model-based risk probability (MPP), patients are stratified into three management categories: low risk (MPP <0.21, regular follow-up), intermediate risk (MPP 0.21–0.87, recommend liver biopsy for definitive guidance), and high risk (MPP >0.87, consider initiating antiviral therapy). CHB, chronic hepatitis B; ALT, alanine aminotransferase; MPP, model prediction probability; G, METAVIR inflammation grade; S, METAVIR fibrosis stage.

Based on these rigorously defined thresholds, we propose a clear clinical decision pathway (**Figure 3**). The patient distribution, prevalence of significant injury, and specific diagnostic performance across these three risk categories (low, intermediate, and high) are summarized in **Table 3**. Patients with a Y-HAL Index < 0.21 (low-risk) are recommended for continued monitoring; those with an Index > 0.87 (high-risk) are strong candidates for initiating antiviral therapy; and for patients in the intermediate “grey zone” (Index 0.21-0.87, representing 38.5% of the cohort), a liver biopsy is recommended to guide treatment decisions.

**Table 3:** Risk stratification and diagnostic performance of the Y-HAL Index in the total cohort.

Risk category (Y-HAL index)	Patient distribution N (%)	Prevalence of significant injury N (%)	Sensitivity (%)	Specificity (%)	PPV (%)	NPV (%)
Low Risk (< 0.21)	118/423 (27.9%)	7/118 (5.9%)	97.10%	–	–	94.10%
Intermediate Risk (0.21 - 0.87)	163/423 (38.5%)	102/163 (62.6%)	N/A	N/A	N/A	N/A
High Risk (> 0.87)	142/423 (33.6%)	133/142 (93.7%)	–	95.00%	93.70%	–

Significant histological injury was defined as METAVIR necroinflammation grade ≥G2 and/or fibrosis stage ≥S2. PPV, positive predictive value; NPV, negative predictive value. Intermediate-risk patients are recommended for liver biopsy to definitively guide clinical management.

## 4 Discussion

This nationwide multicenter biopsy-based study uncovers a critical clinical reality: among treatment-naïve CHB patients aged ≤ 30 years with persistently normal ALT, a substantial proportion harbors significant histological liver injury. Our findings show that 48.0% of these patients exhibit significant necroinflammation (≥G2) and 48.5% having significant fibrosis (≥S2). This high prevalence aligns with and expands upon emerging evidence (Jiang et al., 2023; Li et al., 2023), challenging the traditional paradigm that relies on normal ALT as a reliable surrogate for intrahepatic disease quiescence. Crucially, our analysis provides histological confirmation that in this young cohort, ALT levels even within the normal range fail to reflect the underlying liver pathology, highlighting a “gray zone” where progressive disease may be systematically undertreated.

To address this diagnostic dilemma, we developed and validated the Y-HAL model as a pragmatic non-invasive tool to identify high-risk individuals. The strength of the model lies in its integration of three routinely available parameters: anti-HBc, LSM, and AST.

A high anti-HBc level signifies an intensified host immune response associated with immune-mediated hepatocyte injury (Lazarevic et al., 2023; Shi et al., 2023). LSM directly quantifies hepatic fibrosis as a surrogate for structural damage (Tapper and Parikh, 2023). Notably, despite being within the normal range, AST demonstrated a robust predictive value, likely due to its distinct pathophysiological profile. Unlike ALT, which is primarily cytoplasmic, approximately 80% of AST is located within mitochondria. Therefore, mitochondrial damage during chronic immune attacks often leads to subtle AST elevations before massive cell membrane rupture occurs (which would elevate ALT). Furthermore, the half-life of AST in circulation is approximately 17 hours, significantly shorter than the 47 hours of ALT, allowing AST to reflect recent inflammatory activity and metabolic stress more sensitively (Giannini et al., 2005). The Y-HAL model synergistically integrates these indicators of immune activity (anti-HBc), structural alteration (LSM), and subcellular leakage (AST) into a robust composite indicator.

Our results are strongly corroborated by a growing body of literature questioning the safety of deferring treatment in young, ALT-normal CHB patients. For instance, Wu et al. and Feng et al. both reported high rates of significant histological injury (54.6% and 78%, respectively) in ALT-normal patients, emphasizing the inadequacy of ALT alone in excluding active disease (Wu et al., 2019; Feng et al., 2022). Similarly, studies focusing on HBeAg-positive, ALT-normal patients found that 57.5% had significant histological changes, with ALT levels as low as >20 U/L being an independent risk factor (Duan et al., 2024). Our study consolidates these findings by providing definitive, multi-center histological evidence specifically in patients under 30, a demographic traditionally considered to be in a benign “immune-tolerant” phase.

Compared to 12 established non-invasive models, including FIB-4 and APRI, the Y-HAL model demonstrated superior discriminative ability (AUC: 0.918). Conventional tools were primarily derived from older cohorts with elevated liver enzymes (Castera et al., 2025), and their inherent reliance on age and ALT renders them inadequately sensitive in young, ALT-normal patients. By bypassing these fundamental drawbacks, Y-HAL provides a more tailored assessment.

To facilitate proactive clinical management, our tripartite pathway stratifies patients into low-risk (monitoring), intermediate-risk (biopsy), and high-risk (antiviral therapy) categories. By applying stringent cut-offs, this framework safely minimizes unnecessary biopsies while accurately identifying individuals who urgently require treatment. International guidelines provide limited and conservative recommendations for young, ALT-normal CHB patients, often advocating for observation rather than treatment. This creates a significant “gray zone” where progressive liver injury may go undetected and untreated (Ma et al., 2025). Recent years have witnessed increasing calls for expanding antiviral treatment indications in CHB, driven by the recognition that a substantial proportion of patients outside traditional treatment criteria experience disease progression (Chen and Lau, 2024; Lin and Kao, 2024; Zhang et al., 2024). The updated 2024 WHO guidelines further underscore this paradigm shift, advocating for lower HBV DNA and ALT thresholds to simplify and broaden treatment access (Wong and Lemoine, 2025). Our study provides timely and powerful support for this evolution. By offering a validated, practical tool for precise risk stratification, the Y-HAL model enables clinicians to move beyond a uniform “watchful waiting” approach. It empowers earlier intervention in high-risk young patients, potentially halting insidious fibrosis progression and reducing the long-term burden of HBV-related complications, thereby aligning with the global strategic goal of eliminating viral hepatitis as a public health threat by 2030.

Several limitations of our study warrant careful consideration. First, the cross-sectional study design identifies prevalent injury but does not allow for definitive conclusions regarding long-term clinical outcomes or the direct longitudinal benefit of early antiviral therapy initiation. Prospective longitudinal studies are required to confirm if early intervention based on Y-HAL stratification leads to improved prognosis. Second, a potential selection bias exists, as the cohort only included patients willing and eligible to undergo liver biopsy. Patients who declined the procedure were excluded, which may result in injury prevalence estimates that differ from the broader clinical population. Third, regarding the HBeAg-negative subgroup, our preliminary analysis suggested that liver injury severity was independent of HBeAg status. However, the small sample size of this subgroup (N = 40) introduces a risk for Type II error. Because HBeAg-negative CHB can involve complex viral mutations (Tacke et al., 2004), we cannot rule out distinct occult pathological features, and these results should be interpreted cautiously until confirmed by larger studies. Fourth, despite the multicenter design involving nine hospitals, potential center effects cannot be entirely excluded. While standardized protocols for data collection and biopsy procedures were strictly implemented across all centers to minimize inter-institutional variability, and histological assessments were centrally performed by blinded expert pathologists, minor variations in local patient demographics or clinical practices might still exist. Future studies with larger, more diverse cohorts are needed to further evaluate the consistency of the Y-HAL model across different healthcare settings. Finally, while Y-HAL performed well in our internal validation set (AUC: 0.929), the lack of external validation across diverse healthcare systems or ethnic populations limits its immediate generalizability. Further validation in independent external cohorts is essential before recommending its widespread implementation in routine clinical workflows.

## 5 Conclusion

In summary, this large-scale biopsy-based study definitively demonstrates that a substantial proportion of young chronic hepatitis B patients with persistently normal ALT harbor significant histological injury, undetectable by conventional biomarkers. The novel Y-HAL model, incorporating anti-HBc, LSM, and AST, provides an accurate and practical tool for identifying these high-risk individuals. By enabling early and precise risk stratification, the Y-HAL model facilitates a necessary paradigm shift in clinical practice, moving beyond the sole reliance on ALT toward a proactive and personalized management strategy for young CHB patients.

## Data Availability

The original contributions presented in the study are included in the article/**Supplementary Material**. Further inquiries can be directed to the corresponding author.

## Glossary

AASLD: American Association for the Study of Liver Diseases
AFP: α-fetoprotein
ALB: albumin
ALP: alkaline phosphatase
ALT: alanine aminotransferase
APASL: Asian Pacific Association for the Study of the Liver
ART: antiviral therapy
AST: aspartate aminotransferase
AUC: area under the curve
BMI: body mass index
CHB: chronic hepatitis B
EASL: European Association for the Study of the Liver
GGT: γ-glutamyl transpeptidase
GLB: globulin
HB: hemoglobin
HBcAb: anti-hepatitis B core antibody
HBV: Hepatitis B virus
HBsAg: hepatitis B surface antigen
HCV: hepatitis C virus
HCC: hepatocellular carcinoma
HDV: hepatitis D virus
HEV: hepatitis E virus
HIV: human immunodeficiency virus
INR: international normalized ratio
IQR: interquartile ranges
LB: liver biopsy
LSM: liver stiffness measurement
MASLD: metabolic dysfunction-associated steatotic liver disease
Neu: Neutrophils
PLT: platelet count
PTA: prothrombin time activity
ROC: receiver operating characteristic
TBIL: total bilirubin
VIF: variance inflation factor
WBC: white blood cell count.
