# Supplementary material for: Normal ALT masks occult liver injury in young chronic hepatitis B patients: a call for early treatment intervention
Source: Front Cell Infect Microbiol. 2026 Jun 2;16:1845162. doi: 10.3389/fcimb.2026.1845162 (PMC13269269; doi:10.3389/fcimb.2026.1845162)
Supplement: Supplementary file 1 [file Table1.docx]

**Supplementary Material**

Supplementary Table 1. Characteristics of Patients with Significant Histological Injury (G≥2 or S≥2), Stratified by HBeAg Status.

Supplementary Table 2. Univariate and Multivariate Logistic Regression Analysis for Predicting Significant Histological Injury in the Training Cohort.

Supplementary Table 3. Cutoff values and performance of the model in the training set and validation set.

Supplementary Table 4. AUROCs of the noninvasive models for identifying moderate inflammation or significant fibrosis in the total people.

Supplementary Figure 1. Patient Enrollment Flowchart.

Supplementary Figure 2. Distribution of histological injury in the study cohort.

Supplementary Figure 3. Proportion of significant histological injury stratified by HBeAg status

Supplementary Figure 4. ROC curves of the Y‑HAL model in the training and validation Sets.

Supplementary Figure 5. Calibration curves of the Y-HAL model for predicting significant histological injury.

**Supplementary Table 1. Characteristics of Patients with Significant Histological Injury (G≥2 or S≥2), Stratified by HBeAg Status.**

| Variables | Total  N=242 | HBeAg(-)  N=40 | HBeAg(+)  N=202 | *P* value |
| --- | --- | --- | --- | --- |
| Age,y | 29.0 [27.0-29.0] | 29.0 [28.0-29.0] | 29.0 [27.0-29.0] | 0.363 |
| Male gender,n(%) | 87 (36.0%) | 17 (42.5%) | 70 (34.7%) | 0.445 |
| WBC(10^9^/L) | 5.94 [5.04-7.01] | 5.96 [4.92-7.40] | 5.94 [5.06-6.99] | 0.704 |
| RBC(10^9^/L) | 4.84 [4.46-5.19] | 4.90 [4.41-5.18] | 4.84 [4.48-5.20] | 0.681 |
| HGB(g/dL) | 14.5 [13.4-15.6] | 14.6 [13.6-15.6] | 14.5 [13.3-15.6] | 0.639 |
| PLT(10^9^/L) | 201 [170-230] | 202 [162-228] | 201 [170-230] | 0.717 |
| AST(U/L) | 25.1 [20.0-32.0] | 21.2 [18.8-29.5] | 26.0 [20.9-33.0] | 0.053 |
| ALT(U/L) | 28.0 [19.0-37.9] | 25.4 [17.9-36.5] | 28.0 [19.4-38.0] | 0.221 |
| ALB(g/L) | 44.5 [41.8-46.8] | 44.4 [40.7-46.8] | 44.5 [41.9-46.9] | 0.795 |
| GLB(g/L) | 28.0 [25.3-30.9] | 26.8 [25.1-29.8] | 28.2 [25.4-31.1] | 0.097 |
| TB(μmol/L) | 12.4 [9.17-17.2] | 11.1 [8.48-14.3] | 12.4 [9.33-18.2] | 0.129 |
| DB(μmol/L) | 4.30 [3.38-6.35] | 4.00 [3.10-5.37] | 4.30 [3.42-6.70] | 0.124 |
| ALP(U/L) | 65.0 [56.0-84.0] | 61.5 [52.2-80.8] | 65.0 [56.0-84.0] | 0.533 |
| GGT(U/L) | 18.5 [13.0-32.0] | 18.5 [13.8-26.5] | 18.5 [13.0-33.0] | 0.867 |
| PT(s) | 13.2 [12.1-13.8] | 12.6 [11.7-13.5] | 13.2 [12.1-13.9] | 0.045 |
| INR | 1.03 [0.98-1.08] | 1.01 [0.95-1.06] | 1.03 [0.98-1.08] | 0.062 |
| HBsAg,log_10_  (IU/ml) | 3.40 [3.01-3.78] | 3.24 [2.24-3.68] | 3.40 [3.09-3.79] | 0.009 |
| HBsAb,  Positive,n(%) | 21 (8.68%) | 3 (7.50%) | 18 (8.91%) | 1.000 |
| HBeAb,  Positive,n(%) | 135 (55.8%) | 16 (40.0%) | 119 (58.9%) | 0.043 |
| HBcAb,(IU/ml) | 7.00 [3.96-8.27] | 7.00 [5.56-7.04] | 6.99 [3.62-8.60] | 0.426 |
| HBVDNA  log10(IU/ml) | 4.30 [2.93-6.89] | 3.00 [2.27-4.38] | 4.60 [3.23-7.29] | <0.001 |
| AFP(ng/mL) | 2.56 [1.80-4.22] | 2.66 [1.70-4.14] | 2.55 [1.84-4.27] | 0.872 |
| LSM(KPa) | 7.35 [5.60-10.3] | 7.70 [5.30-10.6] | 7.30 [5.60-10.3] | 0.680 |

Data are presented as median [interquartile range] for continuous variables and n (%) for categorical variables. Group comparisons were made using the Mann-Whitney U test for continuous variables and the Chi-square test or Fisher's exact test for categorical variables, as appropriate. A p-value <0.05 was considered statistically significant. Significant histological injury was defined as necroinflammatory activity grade ≥G2 and/or fibrosis stage ≥S2.

**Abbreviations**: HBeAg, hepatitis B e antigen- WBC, white blood cell count- RBC, red blood cell count- HGB, hemoglobin- PLT, platelet count- AST, aspartate aminotransferase- ALT, alanine aminotransferase- ALB, albumin- GLB, globulin- TB, total bilirubin- DB, direct bilirubin- ALP, alkaline phosphatase- GGT, gamma-glutamyl transferase- PT, prothrombin time- INR, international normalised ratio- HBsAg, hepatitis B surface antigen- HBsAb, hepatitis B surface antibody- HBeAb, hepatitis B e antibody- HBcAb, hepatitis B core antibody- HBV, hepatitis B virus- AFP, alpha-fetoprotein- LSM, liver stiffness measurement.

**Supplementary Table 2. Univariate and Multivariate Logistic Regression Analysis for Predicting Significant Histological Injury in the Training Cohort.**

| Variables | Univariate Analysis | | | Multivariate Analysis | | |
| --- | --- | --- | --- | --- | --- | --- |
|  | OR | 95%CI | P value | OR | 95%CI | P value |
| Age | 0.96 | [0.89-1.02] | 0.196 |  |  |  |
| Male gender | 1.24 | [0.77-1.98] | 0.378 |  |  |  |
| WBC | 1.08 | [0.96-1.22] | 0.224 |  |  |  |
| RBC | 0.95 | [0.65-1.38] | 0.781 |  |  |  |
| HGB | 1.00 | [0.99-1.01] | 0.885 |  |  |  |
| PLT | 1.00 | [0.99-1.00] | 0.162 |  |  |  |
| AST | 1.06 | [1.03-1.09] | <0.001 | 1.06 | [1.02-1.10] | 0.009 |
| ALT | 1.01 | [0.99-1.03] | 0.320 |  |  |  |
| ALB | 0.99 | [0.97-1.01] | 0.441 |  |  |  |
| GLB | 1.02 | [1.00-1.05] | 0.077 |  |  |  |
| TB | 1.01 | [0.99-1.02] | 0.302 |  |  |  |
| DB | 1.03 | [0.99-1.08] | 0.135 |  |  |  |
| ALP | 1.00 | [1.00-1.01] | 0.580 |  |  |  |
| GGT | 1.02 | [1.00-1.04] | 0.012 |  |  |  |
| PT | 1.14 | [0.97-1.35] | 0.117 |  |  |  |
| INR | 1.31 | [0.99-1.72] | 0.057 |  |  |  |
| HBsAg | 0.81 | [0.62-1.06] | 0.131 |  |  |  |
| HBsAb Positive | 2.23 | [0.89-6.45] | 0.089 |  |  |  |
| HBeAg Positive | 1.04 | [0.58-1.86] | 0.895 |  |  |  |
| HBeAb Positive | 1.15 | [0.72-1.84] | 0.546 |  |  |  |
| HBcAb | 1.78 | [1.58-2.01] | <0.001 | 1.84 | [1.62-2.14] | <0.001 |
| HBV DNA | 0.93 | [0.84-1.03] | 0.170 |  |  |  |
| AFP | 1.00 | [0.99-1.01] | 0.436 |  |  |  |
| LSM | 1.30 | [1.17-1.44] | <0.001 | 1.38 | [1.20,1.60] | <0.001 |

Logistic regression analysis was performed to identify factors associated with significant histological injury (defined as necroinflammatory activity grade ≥G2 and/or fibrosis stage ≥S2). Variables demonstrating statistical significance (p < 0.05) in the univariate analysis were subsequently included in the multivariate stepwise logistic regression model.

**Abbreviations:** OR, odds ratio- CI, confidence interval- WBC, white blood cell count- RBC, red blood cell count- HGB, hemoglobin- PLT, platelet count- AST, aspartate aminotransferase- ALT, alanine aminotransferase- ALB, albumin- GLB, globulin- TB, total bilirubin- DB, direct bilirubin- ALP, alkaline phosphatase- GGT, gamma-glutamyl transferase- PT, prothrombin time- INR, international normalised ratio- HBsAg, hepatitis B surface antigen- HBsAb, hepatitis B surface antibody- HBeAg, hepatitis B e antigen- HBeAb, hepatitis B e antibody- HBcAb, hepatitis B core antibody- HBV, hepatitis B virus- AFP, alpha-fetoprotein- LSM, liver stiffness measurement.

**Supplementary Table 3. Cutoff values and performance of the model in the training set and validation set.**

| Patients | AUROC  (95%CI) | Cut off  values | Sensitivity  (%) | Specificity  (%) | PPV  (%) | NPV  (%) | Correctly  Classified (%) | Accuracy  (%) |
| --- | --- | --- | --- | --- | --- | --- | --- | --- |
| Training Set | 0.912  （0.897-0.945） | 0.382 | 90.5 | 78.9 | 85.0 | 86.3 | 83.9 | 85.5 |
| Validation Set | 0.929  （0.885-0.974） | 0.464 | 93.2 | 83.0 | 88.3 | 89.8 | 88.1 | 88.9 |
| Total  set | 0.918  (0.892-0.944) | 0.50 | 86.4 | 84 | 87.8 | 82.1 | 84.9 | 85.3 |

The predictive model was developed to identify significant histological injury (defined as necroinflammatory activity grade ≥G2 and/or fibrosis stage ≥S2). The cutoff value represents the optimal probability threshold determined by maximizing the Youden's index. Performance metrics are reported for the training set, an independent validation set, and the combined total cohort.
**Abbreviations:** AUROC, area under the receiver operating characteristic curve- CI, confidence interval- PPV, positive predictive value- NPV, negative predictive value.

**Supplementary Table 4. AUROCs of the noninvasive models for identifying moderate inflammation or significant fibrosis in the total people.**

| Noninvasive  models | AUROC | 95%CI | Noninvasive  models | AUROC | 95%CI |
| --- | --- | --- | --- | --- | --- |
| Y-HAL | 0.918 | 0.892-0.944 | AAF | 0.870 | 0.838-0.903 |
| FIB-4 | 0.597 | 0.543-0.651 | GPR | 0.602 | 0.549-0.656 |
| AAR | 0.548 | 0.493-0.603 | GUCI | 0.624 | 0.571-0.677 |
| APRI | 0.621 | 0.568-0.674 | GqHBsR | 0.582 | 0.527-0.636 |
| Apind | 0.519 | 0.465-0.574 | S index | 0.646 | 0.594-0.698 |
| CDS | 0.575 | 0.522-0.629 | FibroQ | 0.572 | 0.517-0.627 |
| FI | 0.602 | 0.547-0.656 |  |  |  |

AUROCs of various existing noninvasive models for the identification of moderate-to-severe necroinflammatory activity (≥G2) or significant fibrosis (≥S2) within the total study cohort (N=423).

**Abbreviations:** AUROC, area under the receiver operating characteristic curve- CI, confidence interval- FIB-4, Fibrosis-4 index- AAR, AST/ALT ratio- APRI, AST to Platelet Ratio Index- CDS, cirrhosis discriminant score- FI, Fibrosis Index- GPR, gamma-glutamyl transpeptidase to platelet ratio- GUCI, Göteborg University Cirrhosis Index- GqHBsR, Hepatitis B s-antigen quantitative relation- S index, S index- AAF, novel model (from this study)- FibroQ, Fibro-Q score.

**
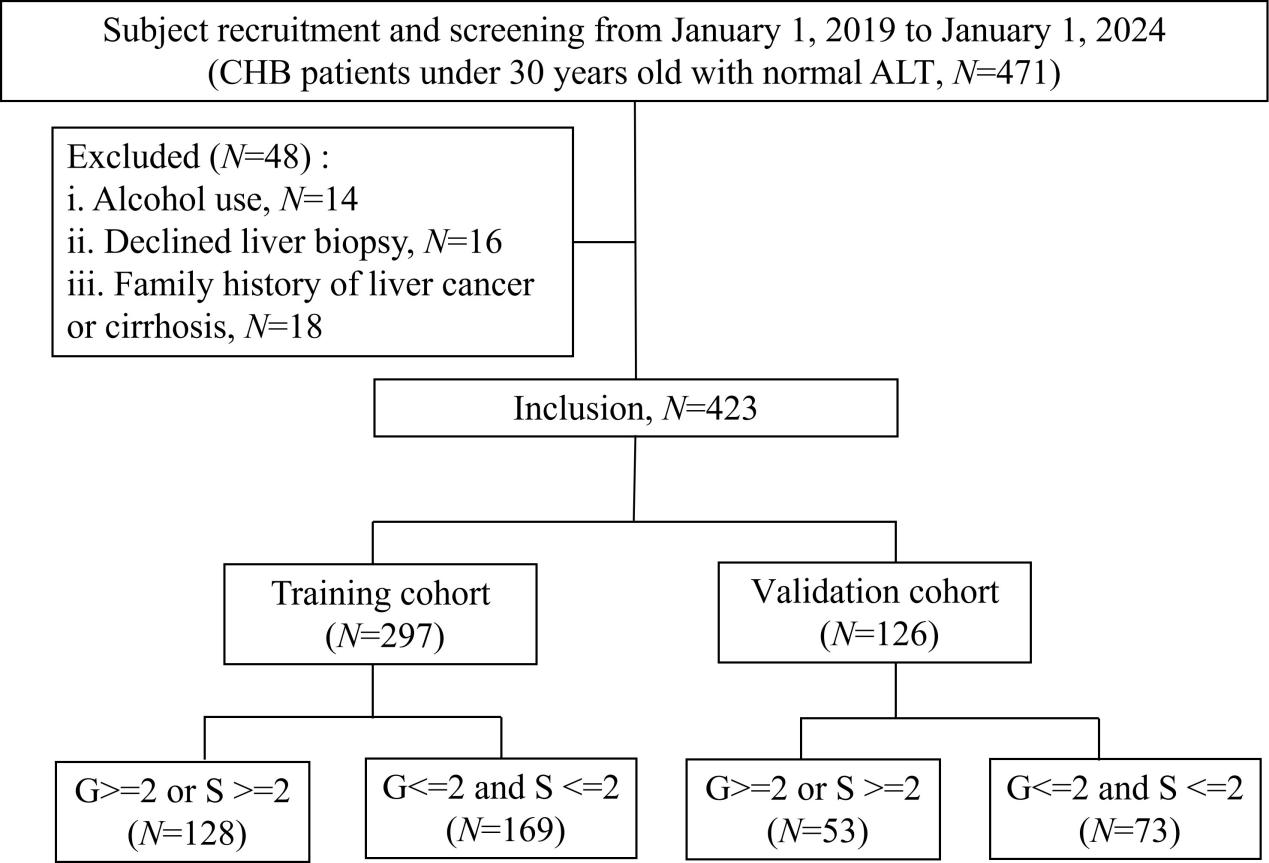
**

**Supplementary Figure 1. Patient Enrollment Flowchart.**

A total of 471 treatment-naïve chronic hepatitis B (CHB) patients aged ≤30 years with persistently normal alanine aminotransferase (ALT) were screened. After exclusions, 423 patients were included and randomly divided into a training cohort (N=297) and a validation cohort (N=126) at a 7:3 ratio. Histological injury was defined as METAVIR necroinflammation grade ≥G2 and/or fibrosis stage ≥S2.

**
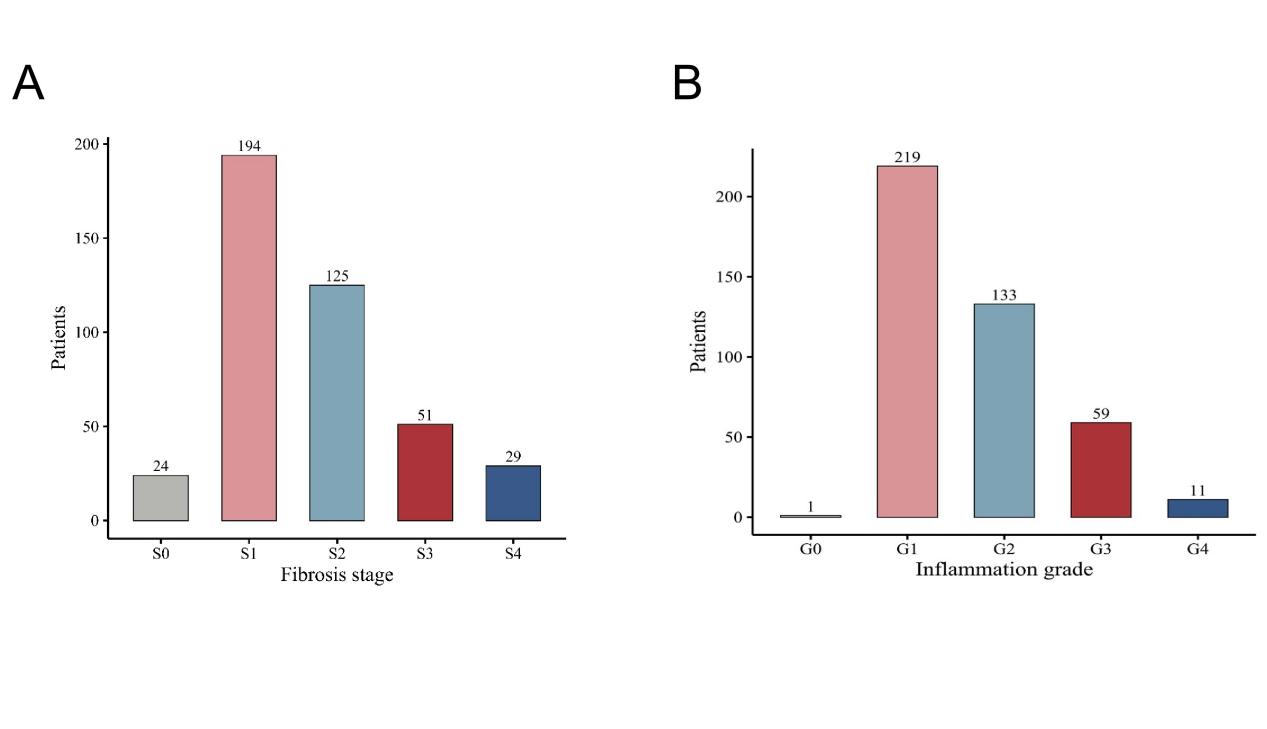
**

**Supplementary Figure 2. Distribution of histological injury in the study cohort.**

(A) Bar chart showing the number of patients across fibrosis stages (S0–S4) as assessed by the METAVIR scoring system. (B) Bar chart showing the number of patients across inflammation grades (G0–G4) as assessed by the METAVIR scoring system.

**
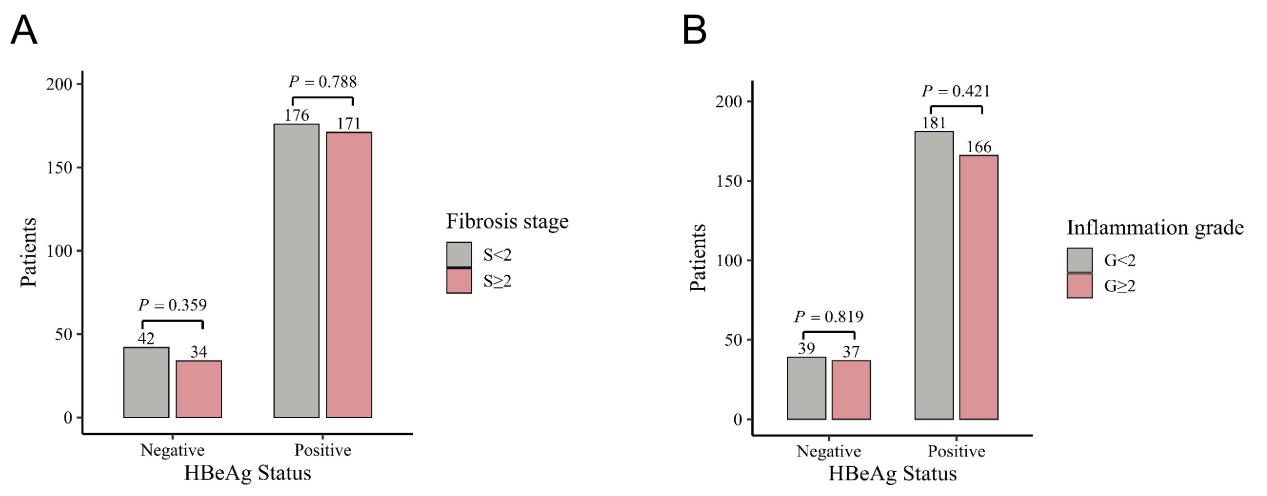
**

**Supplementary Figure 3. Proportion of significant histological injury stratified by HBeAg status.** (A) Inflammation grade (G<2 vs. ≥G2) and (B) fibrosis stage (S<2 vs. ≥S2) distributions in HBeAg-negative and HBeAg-positive subgroups among patients with significant injury. P-values were calculated using the Chi-square test.

**
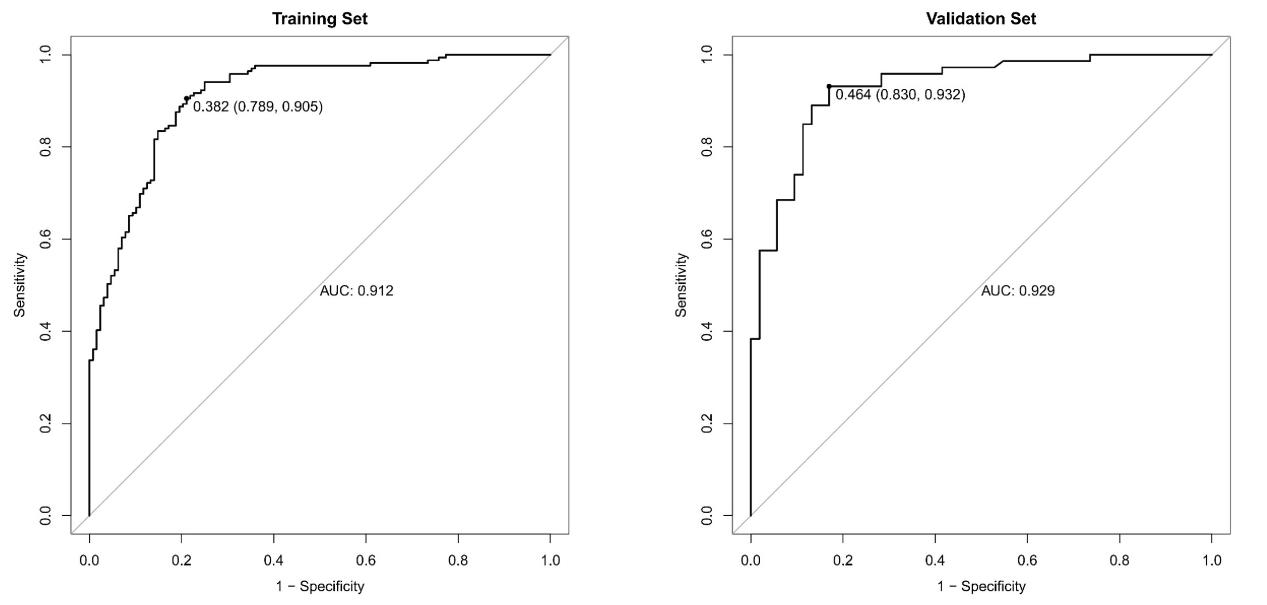
**

**Supplementary Figure 4.ROC curves of the Y‑HAL model in the training and validation Sets.** (A) Training cohort (area under the curve, AUC= 0.912, 95% CI: 0.897–0.945). (B) Internal validation cohort (AUC = 0.929, 95% CI: 0.885–0.974). The optimal cutoff probability (0.464) is indicated, yielding a sensitivity of 93.2% and specificity of 83.0% in the validation set.


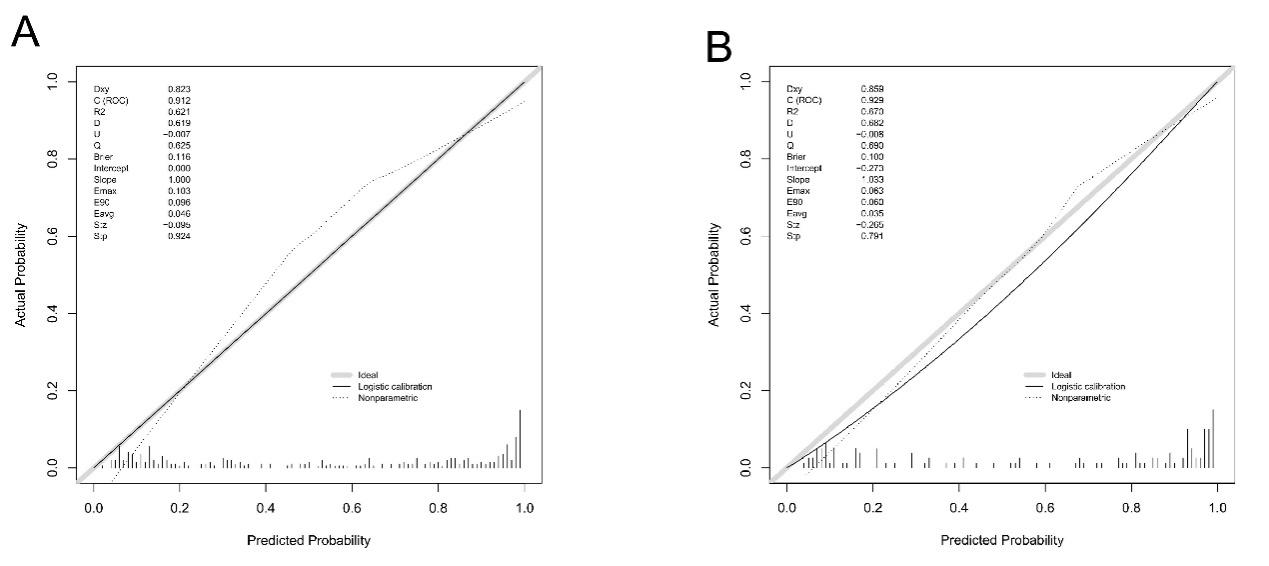


**Supplementary Figure 5. Calibration curves of the Y-HAL model for predicting significant histological injury.**(A) Calibration curve in the training cohort. (B) Calibration curve in the internal validation cohort. The x-axis represents the model-predicted probability, while the y-axis indicates the actual observed probability. The thick grey diagonal line represents the ideal reference line. The solid black line and the dotted line represent the logistic calibration and nonparametric fit, respectively. Goodness-of-fit was evaluated using Spiegelhalter's Z-test. The non-significant P-values in both cohorts (S:p = 0.924 and S:p = 0.791) indicate an excellent calibration.
